# Management of synchronous adenocarcinoma of the esophago-gastric junction and ampulla of Vater: case report of a surgically challenging condition

**DOI:** 10.1186/1754-9493-3-23

**Published:** 2009-09-28

**Authors:** Namita Jayaprakash, Fardod O'Kelly, Kheng Tian Lim, John Vincent Reynolds

**Affiliations:** 1 Department of Clinical Surgery, Trinity Centre, Trinity College Dublin and St James's Hospital, Dublin 8, Ireland

## Abstract

We report herein a case of a synchronous presentation of an adenocarcinoma of esophagago-gastric junction type II and an ampullary tumor that was treated by combined Whipple's pancreaticoduodenectomy, total gastrectomy and esophagectomy. The magnitude of this operation was safely achieved with meticulous surgical techniques and perioperative care without any major short or long term complications. Patient returned to a good quality of life at six-month follow up with no further gastrointestinal symptoms or evidence of disease recurrence.

## Background

There has been an increase in the West in recent years of adenocarcinoma of the esophagus and esophagago-gastric junction. Standard work-up in high volume centers includes computed tomography (CT) scan, endoscopic ultrasound scan (EUS) and [^18^F]-2-fluoro-2-deoxy-D-glucose-positron emission tomography (^18^F-FDG-PET) imaging. Adenocarcinoma of the esophago-gastric junction (AEG) is classified as per Siewert et al. into AEG type I, arising in the distal esophagus usually on a background of Barrett's esophagus, AEG type II which arises at the anatomic cardia, and AEG Type III tumors are sub-cardial gastric carcinomas which invade the junction and lower esophagus from below [1,2]. The surgical approach to AEG type I tumors most usually involves a transthoracic esophagectomy with abdominal and mediastinal lymphadenectomy. For AEG type II and III, a total gastrectomy and transmediastinal lymphadenectomy and distal esophagectomy is increasingly utilized [3]. Multimodal approaches, either chemotherapy alone or combined with radiation therapy prior to surgery are increasingly considered for patients with locally advanced disease [4]. The best 5-year survival for surgery for localized AEG tumors is approximately 40 per cent [5].

Ampullary cancers, unlike pancreatic carcinomas, may present at an early stage. The goal of surgical management, as in all tumors, remains removal of all gross and microscopic disease from the pancreas and draining lymph node basins [6]. The 5-year survival for completely resected localized disease is approximately 40 per cent [7].

In this high volume esophageal (50-80 resections/year) and middle volume pancreatic center (8-15 Whipples/year) a routine work up of a patient with AEG type II junctional tumor uncovered a synchronous ampullary tumor. The case and management considerations are discussed herein.

## Case Presentation

**Figure 1:**
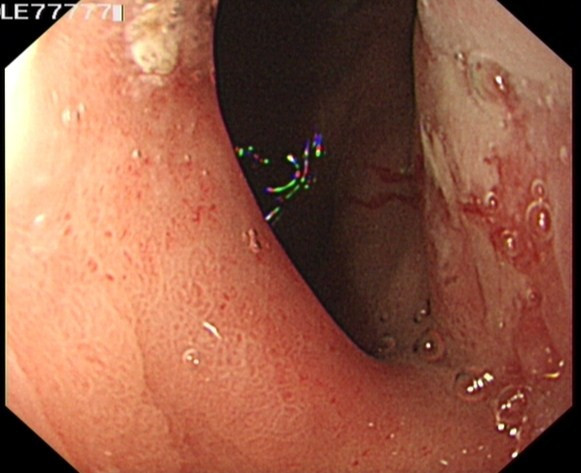
**An upper gastrointestinal endoscopy showed irregular AGE Type II junctional tumour with a tongue of Barrett's esophagus**.

A 62 year old man was referred to our service in 2008 with a history of epigastric pain and melaena of one week duration. An upper gastro-intestinal endoscopy revealed an AEG type II junctional tumour (Figure 1). Biopsies revealed an invasive moderately differentiated adenocarcinoma.

**Figure 2:**
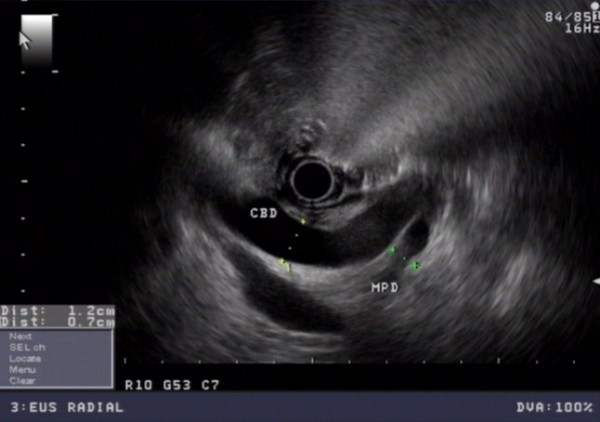
**EUS demonstrated significant dilatation of both the CBD (12 mm in diameter) and MPD (7 mm in distal portion)**.

**Figure 3:**
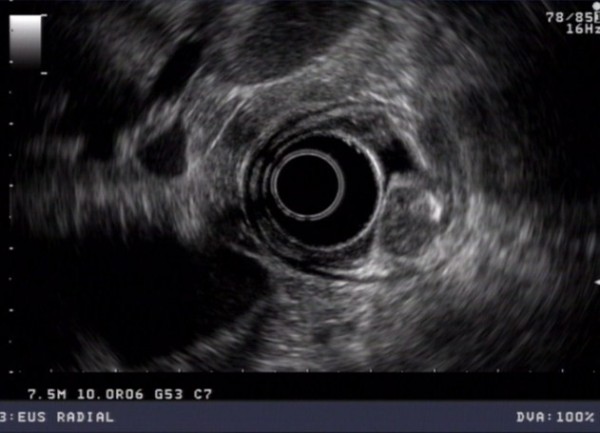
**EUS demonstrated a 12 mm homogeneous ampullary tumour with no significant loco-regional nodes**.

**Figure 4:**
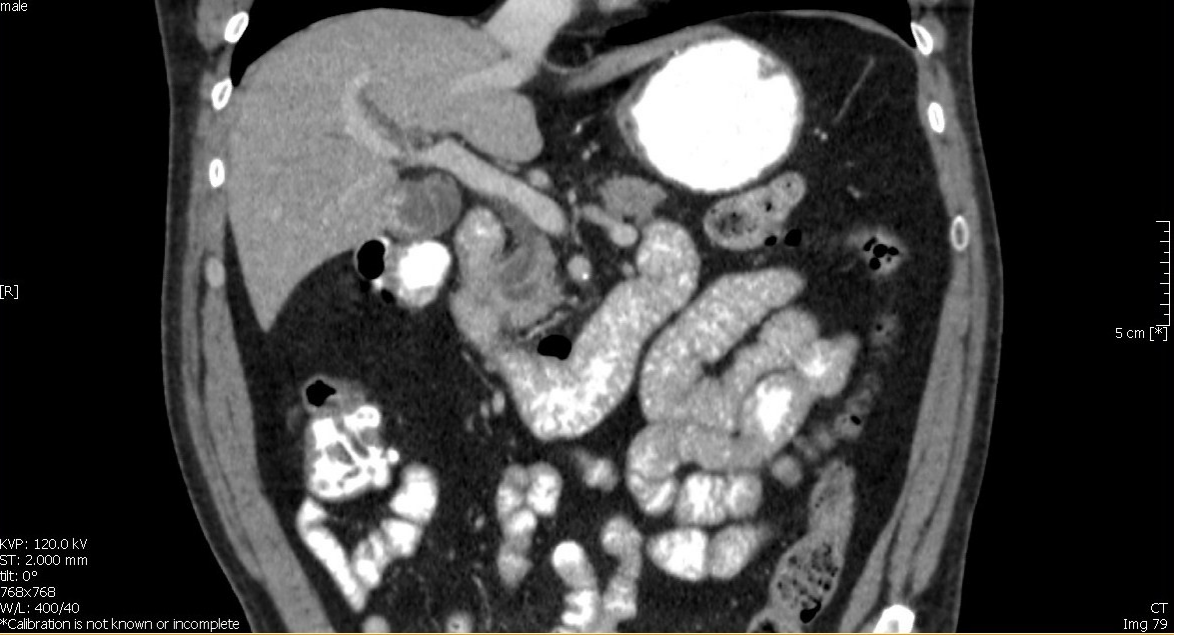
**Abdominal Computed Tomography showed the double duct sign**.

**Figure 5:**
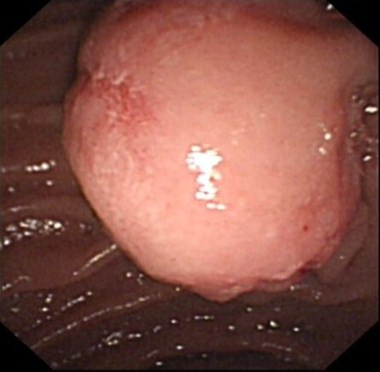
**Endoscopic view during ERCP showed a large ampullary tumour**.

In routine blood tests his liver function tests were noted to be abnormal, with an AST of 25 IU/L and alkaline phosphatase of 201 IU/L. Endoscopic ultrasound revealed a uT2N0 tumor at the cardia, with one indeterminate peritumoral node. There was also significant dilatation of the common bile duct (CBD) at the duodenal ampulla and major pancreatic duct (MPD) (Figure 2). An intra-ampullary homogenous tumor was identified confined to the ampulla of Vater (Figure 3). CT imaging revealed no evidence of either an esophageal or ampullary tumor, but there was a double-duct sign of biliary and pancreatic duct dilatation (Figure 4). ^18^F-FDG-PET imaging revealed no abnormal uptake. Endoscopic Retrograde Cholangiopancreatography (ERCP) revealed a tumor with a large distorted ampulla (Figure 5) and biopsies revealed high grade dysplasia and intra mucosal carcinoma.

The patient had good overall performance status. He had a background history of insulin dependent diabetes mellitus with secondary complications including retinopathy and peripheral neuropathy. His HbA1c on admission was 8.0 g/dL. He also had a history of ischemic heart disease with subsequent angioplasty in 2006. He had hypertension, diverticular disease and colonic polyps. A pre-operative exercise stress test and ECHO revealed good functional capacity with an ejection fraction of 60-65%. His FEV1 was 2.37 litres and his FVC was 4.30 litres. Pre-assessment by the anaesthetist, cardiologist and endocrinologist certified patient fit for major operation.

**Figure 6:**
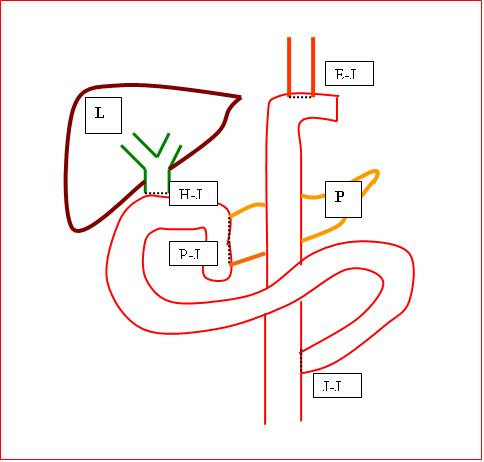
**A drawing illustrating the liver (L), pancreas (P), hepatico-jejunostomy (H-J), pancreatico-jejunostomy (P-J), esophago-jejunostomy (E-J) and jejuno-jejunostomy (J-J) anastomoses**.

The decision was made to proceed to synchronous resections with a planned Whipple's, total gastrectomy and distal esophagectomy, mediastinal dissection and Roux-en-Y esophago-jejunal reconstruction in that order respectively via the upper midline laparotomy incision. There was no technical difficulties in reconstructing the four anastomoses namely hepatico-jejunostomy, pancreatico-jejunostomy, esophago-jejunostomy and jejuno-jejunostomy (Figure 6). The operation was uneventful, taking 340 minutes, the blood loss was 620 mls, and he was extubated immediately postoperatively. The patient spent 10 days post operatively in the High Dependency Unit (HDU). Analgesia was provided via an epidural at the level of T7/T8 and the patient was given total parenteral nutrition (TPN). He experienced no major complications and was discharged home 22 days postoperatively.

Histology of the junctional tumor revealed a 6 mm tumor, pT1 N0 Mx, with 0 out of 16 nodes involved and negative margins. The ampullary tumor was invasive adenocarcinoma, pT1 N0 Mx, and 0 out of 7 nodes were involved. No adjuvant therapy is being considered. Patient returned to a good quality of life at six-month follow up with no further gastrointestinal symptoms or evidence of disease recurrence.

## Discussion

Synchronous presentation of esophago-gastric and peri-ampullary adenocarcinoma is extremely rare. In a study by Lee et al., 3.4% of gastric cancers were associated with synchronous tumors, most commonly colorectal cancer [7]. Another review of 10,090 patients with gastric cancer reported that 96 had synchronous tumors and 5 of those were pancreatic carcinoma [8]. The management of a case of squamous cell carcinoma (SCC) of the esophagus with an intraductal pancreatic polypoid tumor has been reported, as well as a case of SCC of the esophagus with adenocarcinoma of the ampulla of Vater [9,10]. There was a report on technical challenges in avoiding injury to the middle colic vessels with successful pancreaticoduodenectomy for carcinoma of the ampulla of Vater after esophagectomy with remnant gastrectomy 17 years prior [11]. To our knowledge there is no reported case of synchronous adenocarcinoma of the esophago-gastric junction and ampullary adenocarcinoma.

For both tumors independently, surgical resection is the only curative approach, and the risk of mortality for each procedure should be less than 5 per cent in high-volume centers. Neoadjuvant chemotherapy or chemoradiotherapy for AEG type II junctional adenocarcinoma was discussed but not pursued since this appeared localized and node-negative with a high probability of an R0 resection [12]. The role of perioperative chemotherapy based on MAGIC trial probably offer little benefit in altering resectability, progression-free survival and overall survival of this early stage AEG type II junctional tumour [13]. The risk of adverse effects of haematological and non-haematological toxicity associated with perioperative chemotherapy was considered and discussed.

The surgical treatment options considered in our patient were combined Whipple's pancreaticoduodenectomy, total gastrectomy, distal esophagectomy and Roux-en-Y esophago-jejunal reconstruction along with hepatico-jejunostomy, pancreatico-jejunostomy and jejuno-jejunostomy. We were prepared to do a thoracotomy to get adequate clearance if required, but splitting the diaphragm and a mediastinal dissection and anastomosis were achievable via the abdominal incision. Increasing evidence supports the rationale to avoid transthoracic surgery for AEG type II tumors which are predominantly of gastric origin and rarely involve high mediastinal or sub-carinal nodes [1,3].

For the ampulla, the EUS and ERCP were more in keeping with an invasive rather than an in situ cancer, and a local resection was not considered. A pylorus -preserving approach with gastric preservation combined with a proximal gastrectomy and esophagectomy would have been technically possible, but functional results from total gastrectomy with Roux-en-Y esophago-jejunal reconstruction may be better due to the risk of reflux esophagitis. There appears to be only one previous report in the literature of a similar procedure for synchronous tumors. Mafune et al. described a case of subtotal esophagectomy for SCC, pancreaticoduodenectomy with lymph node dissection and total residual gastrectomy in a patient who had a previous gastrectomy [10].

Synchronous resection of AEG type II junctional tumour and ampullary tumour appears attractive although it takes longer to perform, the benefits are having one general anaesthesia for four anastomotic reconstructions and thus avoiding a second major operation. Whilst a staged procedure may take shorter to perform, the risks are two general anesthesia with potential adhesions complicating second stage procedure and the potential delay in resection of second rapidly progressing tumour. Suzuki et al. reported simultaneous resection of synchronous esophageal and extraesophageal carcinomas can be safely performed with no significant difference in morbidity and mortality compared to solitary esophageal cancer resection as long as complete clearance of both tumours is achieved for favourable long-term results [14].

## Conclusion

This is the first report of combined Whipple's and esophago-gastrectomy for these synchronous tumors. The magnitude of this operation was safely achieved with meticulous surgical techniques and perioperative care without any major complications.

## Consent

Written informed consent was obtained from the patient for publication of this case report and any accompanying images. A copy of the written consent is available for review by the Editor-in-Chief of this journal.

## Competing interests

The authors declare that they have no competing interests.

## Authors' contributions

NJ and FO drafted the manuscript. KTL prepared the figures, reviewed and amended and finalized the manuscript. JVR critically reviewed, amended and finalized the manuscript. All authors read and approved the final manuscript.

## References

[B1] Siewert JR, Feith M, Werner M, Stein HJ (2000). Adenocarcinoma of the esophagastric junction: results of surgical therapy based on anatomical/topographical classification in 1002 consecutive patients. Ann Surg.

[B2] Siewert JR, Stein HJ (1998). Classification of adenocarcinoma of the oesophago-gastric junction. Br J Surg.

[B3] Omloo JM, Lagarde SM, Hulscher JB, Reitsma JB, Fockens P, van Dekken H, Ten Kate FJ, Obertop H, Tilanus HW, van Lanschot JJ (2007). Extended transthoracic resection compared with limited transhiatal resection for adenocarcinoma of the mid/distal esophagus: five-year survival of a randomized clinical trial. Ann Surg.

[B4] Enzinger PC, Mayer RJ (2003). Esophageal Cancer. N Engl J Med.

[B5] Reynolds JV, Muldoon C, Hollywood D, Ravi N, Rowley S, O'Byrne K, Kennedy J, Murphy TJ (2007). Long-term outcomes following neoadjuvant chemoradiotherapy for esophageal cancer. Ann Surg.

[B6] Wray CJ, Ahmad SA, Matthews JB, Lowy AM (2005). Surgery for pancreatic cancer: recent controversies and current practice. Gastroenterology.

[B7] Lee JH, Bae JS, Ryu KW, Lee JS, Park SR, Kim CG, Kook MC, Choi IJ, Kim YW, Park JG, Bae JM (2006). Gastric cancer patients at high risk of having synchronous cancer. World J Gastroenterol.

[B8] Ha TK, An JY, Youn HG, Noh JH, Sohn TS, Kim S (2007). Surgical outcome of synchronous second primary cancer in patients with gastric cancer. Yonsei Med J.

[B9] Kurosaki I, Hatakeyana K, Nihei K, Suzuka T, Tsukada K (2000). Thoracic esophagectomy combined with pylorus preserving pancreaticoduodenectomy in one-stage procedure: report of a case. Surg Today.

[B10] Mafune K, Tanaka Y, Ma YY, Takubo K (1995). Synchronous cancers of the esophagus and the ampulla of Vater after distal gastrectomy: Successful removal of the esophagus, gastric remnant, duodenum, and pancreatic head. J Surg Oncol.

[B11] Nagano Y, Sekido H, Matsuoi K, Ohtsuki K, Gorai K, Kunisaki C, Ike H, Imada T, Shimada H (2005). Successful pancreatoduodenectomy for carcinoma of the ampulla of vater after esophagectomy with remnant gastrectomy. Hepatogastroenterology.

[B12] Murphy TJ, Ravi N, Reynolds JV (2008). Treatment options for esophageal cancer. Expert Opinion Pharmacother.

[B13] Cunningham D, Allum WH, Stenning SP, Thompson JN, Velde CJ Van de, Nicolson M, Scarffe JH, Lofts FJ, Falk SJ, Iveson TJ, Smith DB, Langley RE, Verma M, Weeden S, Chua YJ, MAGIC Trial Participants (2006). Perioperative chemotherapy versus surgery alone for resectable gastroesophageal cancer. N Engl J Med.

[B14] Suzuki S, Nishimaki T, Suzuki T, Kanda T, Nakagawa S, Hatakeyama K (2002). Outcomes of simultaneous resection of synchronous esophageal and extraesophageal carcinomas. J Am Coll Surg.

